# Effects of synchrotron-based X-rays and gold nanoparticles on normal and cancer cell morphology and migration

**DOI:** 10.1107/S1600577522012024

**Published:** 2023-01-13

**Authors:** Elham Shahhoseini, Masao Nakayama, Vanessa Panettieri, Chris Hall, Bryce Feltis, Moshi Geso

**Affiliations:** a Medical Radiation, RMIT University, 289 McKimmies Road, Bundoora, Victoria 3083, Australia; b Division of Radiation Oncology, Kobe University, 7-5-2 Kusunokicho Chuou-ku, Kobe 650-0017, Japan; c Alfred Health Radiation Oncology, 55 Commercial Road, Melbourne, Victoria 3004, Australia; d ANSTO, Australian Synchrotron, 800 Blackburn Road, Clayton, Victoria 3168, Australia; e Human Bioscience, RMIT University, 289 McKimmies Road, Bundoora, Victoria 3083, Australia; University of Malaga, Spain

**Keywords:** synchrotron-based X-rays, cell migration, microbeam radiation therapy

## Abstract

It has been shown lately that gold nanoparticles (AuNPs) and ionizing radiation (IR) have inhibitory effects on cancer cell migration while having promoting effects on normal cells’ motility. Also, IR increases cancer cell adhesion with no significant effects on normal cells. In this study, synchrotron-based microbeam radiation therapy, as a novel pre-clinical radiotherapy protocol, is employed to investigate the effects of AuNPs on cell migration. Experiments were conducted utilizing synchrotron X-rays to investigate cancer and normal cell morphology and migration behaviour when they are exposed to synchrotron broad beams (SBB) and synchrotron microbeams (SMB). This *in vitro* study was conducted in two phases. In phase I two cancer cell lines – human prostate (DU145) and human lung (A549) – were exposed to various doses of SBB and SMB. Based on the phase I results, in phase II two normal cell lines were studied: human epidermal melanocytes (HEM) and human primary colon epithelial (CCD841), along with their respective cancerous counterparts, human primary melanoma (MM418-C1) and human colorectal adenocarcinoma (SW48). The results show that radiation-induced damage in cells’ morphology becomes visible with SBB at doses greater than 50 Gy, and incorporating AuNPs increases this effect. Interestly, under the same conditions, no visible morphological changes were observed in the normal cell lines post-irradiation (HEM and CCD841). This can be attributed to the differences in cell metabolic and reactive oxygen species levels between normal and cancer cells. The outcome of this study highlights future applications of synchrotron-based radiotherapy, where it is possible to deliver extremely high doses to cancer tissues whilst preserving surrounding normal tissues from radiation-induced damage.

## Abstract 2

The effects of synchrotron-based kilovoltage X-rays on a cell’s morphology and motility are investigated.

## 1. Introduction

Synchrotron-generated X-ray beams represent a valuable tool for radiation biology studies. They can be used in a broad range of bio-medical research fields from soft tissue imaging to radiation therapy (Ventura, 2019 ▸; Pełka, 2008 ▸). Synchrotron radiation (SR) generates high-intensity and coherent X-ray beams with dose rates up to 20000 Gy s^−1^ (Pełka, 2008 ▸). As a comparison an X-ray tube can generate a maximum dose rate of 100 Gy s^−1^ close to the output window (Pełka, 2008 ▸). The brightness of synchrotron X-ray beams makes them suitable for the production of monochromatic beams. After monochromation of the white beam, the intensity is lowered by two to six orders; however, the intensity is still high enough to be suitable for a number of radiobiology studies and it is still higher than that of the polychromatic beam generated by conventional medical X-ray tubes. The natural properties of SR, such as low divergence and associated lateral coherence, make it suitable for generating fine and spatially divided radiation beams such as those used in microbeam radiation therapy (MRT). The combination of high intensity and fine radiation fields enables the delivery of a high dose of radiation in a very small radiation field. This can be as small as 25 µm to the target with a minimum amount of scatter to the surrounding areas (Hall & Lewis, 2019 ▸). Pre-clinical *in vivo* and *in vitro* studies with such irradiation have shown the reduction of normal tissue damage while destroying the cancerous tissue (Bouchet *et al.*, 2013 ▸; Laissue *et al.*, 2001 ▸, 2007 ▸; Smyth *et al.*, 2018 ▸; Yang *et al.*, 2014 ▸). A range of theories have been reported as the cause for the different behaviours of normal and cancer cells. The phenomenon occurs when the cells are irradiated with a combination of high dose rate (HDR) X-rays and a micro-sized beam. For instance, it has been reported that the high tolerance of normal cells exposed to MRT may be attributed to differences in normal and cancer cell migration or motility (Crosbie *et al.*, 2010 ▸). Despite several extensive studies on the effects of MRT on normal and cancer cells and tissues, the direct relationship between *in vitro* and *in vivo* studies remains unclear and the underlying mechanisms which cause these outcomes are not well understood (Engels *et al.*, 2020 ▸).

In this *in vitro* study, we have used the unique properties of SR, *i.e.* high dose rates and low divergency, to enable us to irradiate a small area of monolayer cultured normal and cancer cells. Post-irradiation we studied the cells’ behaviour within the radiation field and compared with that of the cells outside the field. In the micro-beam irradiation experiments our dosimetry results for peak-to-valley dose ratio (PVDR) confirmed that the valley dose is negligible compared with that in the peak. We conducted an innovative micrography method (utilizing SBB with 500 µm width) to visualize the effects of a high-intensity 90 kV X-ray beam on the morphology and motility of cultured cells with and without inclusion of AuNPs. Our results were compared with those from cells irradiated with synchrotron microbeam (SMB). These experiments showed that the changes in cells’ morphology become visible with synchrotron broad beam (SBB) at doses greater than 50 Gy. The inclusion of gold nanoparticles (AuNPs) increases this effect. Interestly, no visible morphological changes were observed in normal cell lines up to 96 h post-irradiation. This may be explained by the differences in the cell metabolic and reactive oxygen species level between normal and cancer cells (Mapuskar *et al.*, 2019 ▸).

## 2. Experimental details

### 2.1. Cell lines

Four different cancer cell lines were used – human prostate epithelial cancer DU145 (ATCC^®^HTB-81^TM^; Manassas, VA, USA), human lung epithelial cancer A549 (ATCC^®^CCL-185^TM^; Manassas, VA, USA), human primary melanoma MM418-C1 (RRID:CVCL_C843, provided by A/Pro. Moshi Geso), and human colorectal adenocarcinoma SW48 (ATCC^®^CCL-231^TM^; Manassas, VA, USA) – along with two different normal cell lines – human epidermal melanocytes HEM (ATCC^®^PCS-200–013^TM^; Manassas, VA, USA) and human primary colon epithelial CCD841 CoN (ATCC^®^CRL-1790^TM^; Manassas, VA, USA). To examine the effects of AuNPs and/or ionizing radiation (IR) on viability, a cell proliferation and viability assay (MTS) was performed. More details on the cell culture and viability assays can be found in our previous publication (Shahhoseini *et al.*, 2019 ▸).

### 2.2. AuNPs preparation and cellular uptake

To prepare a range of concentrations of gold nanoparticles, the original AuNP (Yaphank, NY, USA) solution was diluted using cell culture media. The final concentration was 0.197 mg ml^−1^. To determine the cellular uptake of AuNPs, inductively coupled plasma mass spectrometry (ICP-MS) was performed.

More details on the AuNPs’ preparation protocols and the ICP-MS measurement method are found in our previous publication (Shahhoseini *et al.*, 2019 ▸).

### 2.3. Cell exposure with SR

Cell irradiations were performed at hutch 2B on the Imaging and Medical Beamline (IMBL) at the ANSTO Australian Synchrotron. Two different radiation treatments were applied for all cell types – control groups with no AuNPs and treated groups which were incubated with 1 m*M* AuNPs, having 15 nm diameter, for 24 h prior to irradiation. Cells were placed in 25 cm^2^ (T25) flasks until a monolayer of 80–90% confluency was achieved. The samples were then exposed to radiation doses ranging from 50 to 1000 Gy of synchrotron-based 90 kV X-rays. The width of the radiation field size for SBB was 500 µm. This aimed to resemble the size of a typical scratch made in a migration assay. For the microbeam the collimation was an SMB grid, 25 µm peak area and 175 µm pitch. The irradiated samples were incubated for 24 h after irradiation and gently washed with phosphate buffered saline (PBS) to remove the dead cells from the exposed areas. The samples were then observed using a live image microscope for 96 h.

### 2.4. Radiation setup

**Figure 1:**
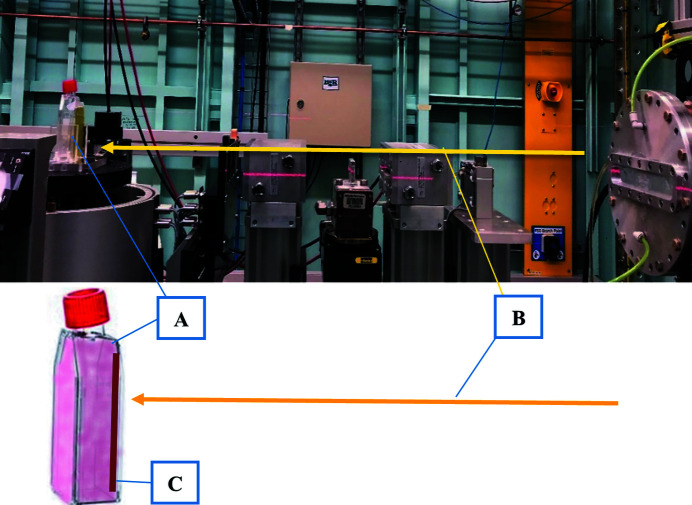
Setup for irradiating the cell samples with kilovoltage SR beams. (A) 25 cm^2^ flask filled completely with culture media, (B) SR (broad beam), (C) the cells attached to the flask’s internal surface.

**Figure 2:**
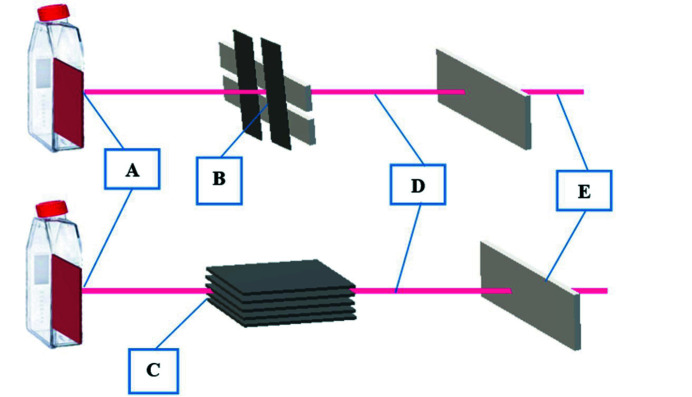
Schematic diagram of the irradiation setup for SBB and SMB. (A) Cell sample, (B) broad beam slit, (C) microbeam collimator, (D) synchrotron pink-beam, (E) beryllium window.

As the SR beam orientation is naturally horizontal, the cells samples had to be placed vertically, perpendicular to the radiation beam. To avoid the cells being out of the culture media during the irradiation time, the flasks were completely filled with media and placed against the radiation beam as shown in Fig. 1 ▸. The flasks were ∼34 m from the source of radiation in hutch 2B at IMBL. In this study, an SBB was arranged by passing the SR beam through a tungsten carbide slit of width 500 µm and height 2 mm. The SMB was produced by passing the SR beam through a multi-slit collimator which formed microbeams with 25 µm width and 500 µm pitch (Stevenson *et al.*, 2017 ▸). Fig. 2 ▸ shows a schematic diagram of the radiation setup for SBB and SMB.

**Figure 3:**
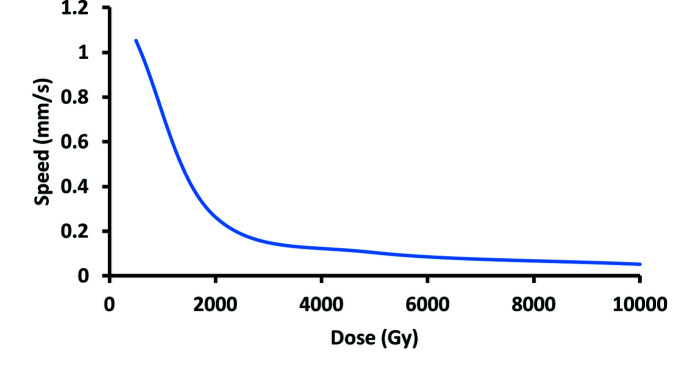
Relationship between the vertical speed of the sample (mm s^−1^) and delivered dose (Gy).

The samples were placed on a motion stage which sets the flasks in the beam. The flasks were moved from top to bottom through the beam to expose the confluent area of the flask to the beam. The delivered dose was calculated based on the measured dose rate of 261.54 Gy s^−1^ and the sample vertical speed. Fig. 3 ▸ shows the relationship between the delivered dose and the speed of the sample, where

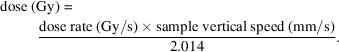




### 2.5. Dose distribution and validation

The dose distribution for both SBB and SMB was measured using a PTW microDiamond detector (Damodar *et al.*, 2018 ▸) and was verified using GAFchromic^TM^ HD-V_2_ films. The films were placed on the surface of the 25 cm^2^ flasks prior to irradiation.

#### 2.5.1. Dose distribution in SBB measured with microDiamond detectors and GAFchromic^TM^ HD-V2 films

**Figure 4:**
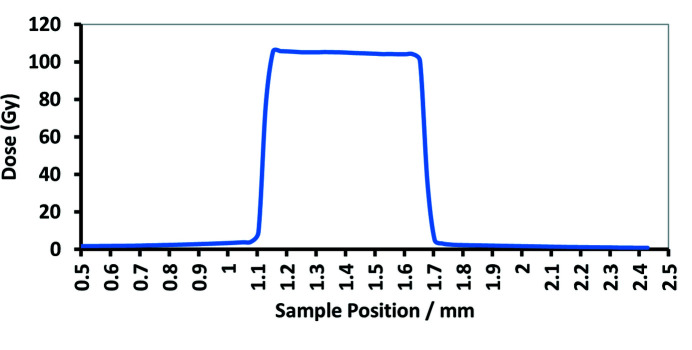
Dose distribution in SBB measured by IMBL PTW microDiamond. The delivered dose was 100 Gy, and the radiation field was 500 µm.

The original dose measurement was performed by the IMBL team using a PTW microDiamond detector and calibrated electrometer. This detector measures the dose in an area of 0.5 mm × 2 mm at 20 mm depth of the sample. Based on this measurement, dose profiles were plotted. As seen in Fig. 4 ▸, the dose out of the radiation field drops dramatically. About 50 µm away from the radiation field the dose is negligible. To verify the delivered dose during irradiation, GAFchromic^TM^ HD-V2 films were used.

**Figure 5:**
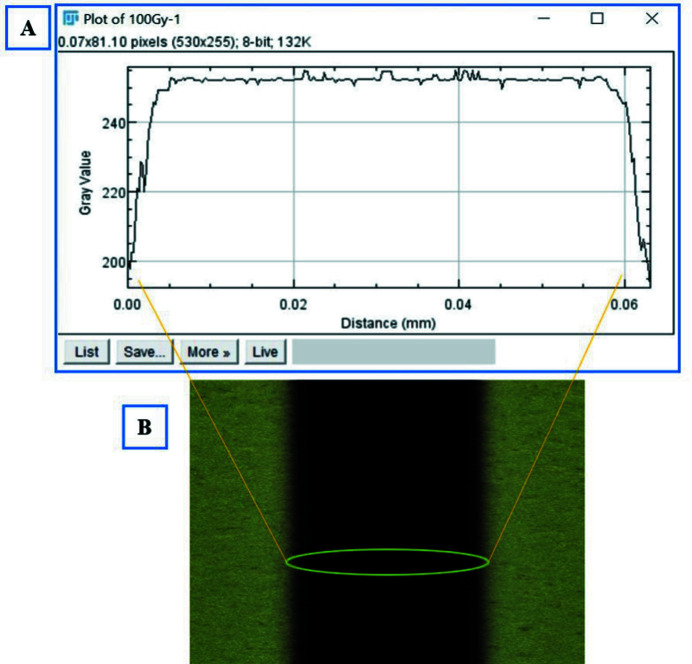
Radiation field exposed to 100 Gy of 90 kV SBB. (A) Dose profile plotted by *ImageJ* ^©^ and (B) GAFchromic^TM^ HD-V_2_ film.

The distribution of the radiation dose was checked using *ImageJ* ^©^ software (Rasband, 1997–2018 ▸) to quantify the optical density of the film which is directly correlated with the dose. The pixel values were plotted against position. Shown in Fig. 5 ▸ are the dose/darkness profiles plotted using *ImageJ* ^©^ for a typical GAFchromic^TM^ film exposed to 100 Gy SBB radiation dose.

#### 2.5.2. Dose distribution in SMB measured with microDiamond detectors and GAFchromic^TM^ HD-V2 films

**Figure 6:**
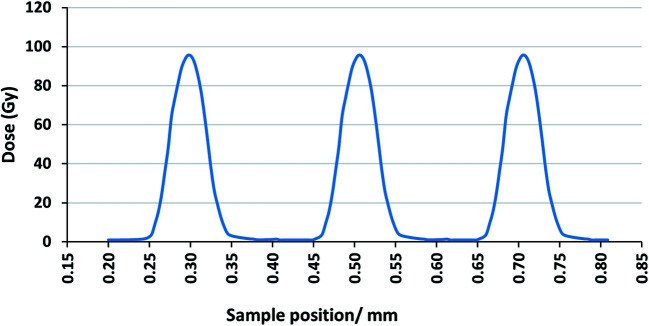
Dose distribution in SMB measured by IMBL PTW microDiamond. The delivered dose was 100 Gy with peak (25 µm) and valley (175 µm).

**Figure 7:**
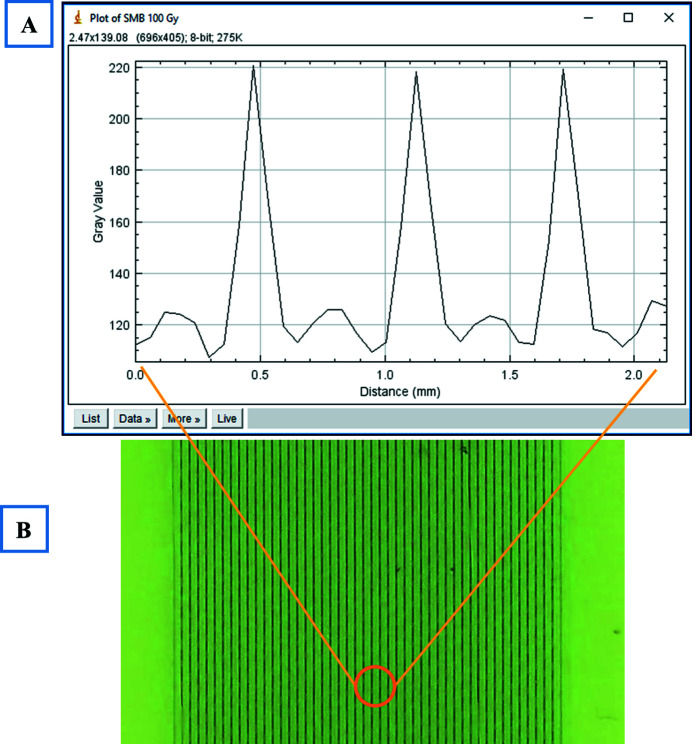
Radiation field exposed to 100 Gy of 90 kV SMB. (A) Dose profile plotted by *ImageJ* ^©^ and (B) GAFchromic^TM^ HD-V_2_ film.

The same procedure was followed for the SMB irradiations. Fig. 6 ▸ shows the dose distribution measured by the PTW microDiamond detector. The plot includes three typical peaks (25 µm) and valley (175 µm) areas. The distribution of the radiation dose was verified using *ImageJ* ^©^ software and the darkness of the film (directly correlated to the dose) was plotted against the distance. Fig. 7 ▸ shows the dose/darkness profile plotted by *ImageJ* ^©^ for typical GAFchromic^TM^ files exposed to 100 Gy SMB radiation dose.

## 3. Results

Cell irradiation with SBB and SMB at the Australian Synchrotron were conducted in two phases. In phase I, two different cancer cell lines, *i.e.* human lung (A549) and human prostate (DU145), with and without AuNPs treatment, were irradiated with 90 kV beam with doses ranging from 50 to 1000 Gy. Based on these results, in phase II we conducted similar experimental protocols to irradiate two different normal cell lines: human epidermal melanocyte (HEM) and human primary colon epithelial (CCD841) The results were compared with their cancerous counterparts: human primary melanoma (MM481) and human colorectal adenocarcinoma (SW48), respectively. In this section, results for all cell types irradiated with SBB are presented, followed by SMB irradiation of the same cells.

### 3.1. Human lung cancer cells (A549) irradiated with SBB

**Figure 8:**
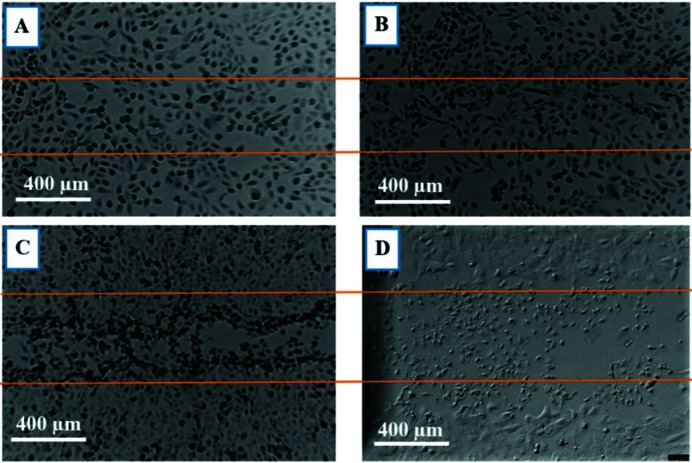
Human lung cancer A549 monolayer cells 24 h after being exposed to SBB with field size of 500 µm. The radiation fields are marked between orange lines: (A) 50 Gy, (B) 100 Gy, (C) 500 Gy and (D) 1000 Gy.

Monolayers of A549 cells grown in T25 flasks were exposed to SBB (width: 500 µm) and SR beam of various doses, *i.e.* 50, 100, 500 and 1000 Gy. To observe radiation-induced morphological changes in cells, the samples were incubated for 24 h after the irradiation, and then washed with PBS to remove dead cells from the culture medium. As seen in Fig. 8 ▸, no visible changes were observed in the cells irradiated with 50 and 100 Gy of SBB beams. Cells located in the radiation field exposed to 500 Gy, Fig. 8(C), show morphological changes. Based on these results, a dose of 1000 Gy was chosen to conduct the rest of the experiments. The boundaries of the irradiated area are marked with orange lines in the following figures. The flask surface is almost covered with damaged cells. Despite PBS wash, most of them are still attached to the polystyrene surface of the T25 flasks. Cells irradiated with 1000 Gy were damaged and killed, and after PBS wash some of the dead cells were removed from the exposed area in a way that still no clear gap is seen in the image, Fig. 8(D).

**Figure 9:**
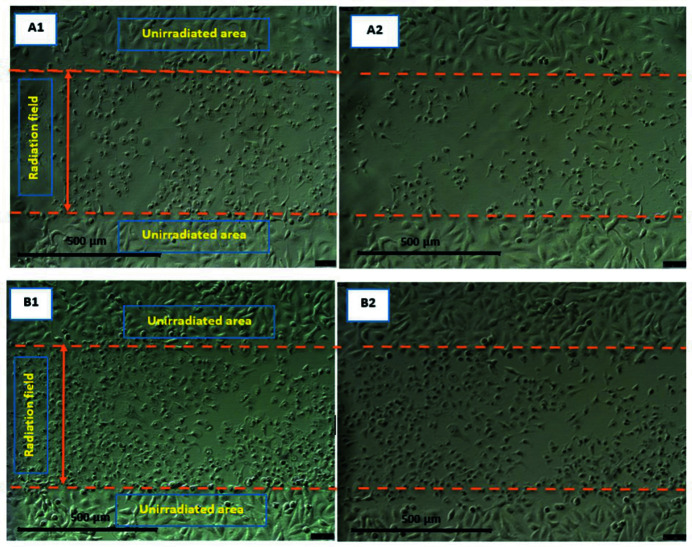
Human lung A549 cancer cells irradiated with 1000 Gy SBB. The radiation field is marked by orange dotted lines. (A1) Control (no AuNPs) 24 h, (A2) control (no AuNPs) 48 h, (B1) treated group (with 1 m*M* AuNPs) 24 h and (B2) treated group (with 1 m*M* AuNPs) 48 h post-irradiation with PBS wash.

As seen in Fig. 9 ▸, A549 cells in two groups, *i.e.* control and treated with 1 m*M* AuNPs, were irradiated with 1000 Gy SBB and observed for morphological changes over time post-irradiation.

### 3.2. Human prostate cancer cells (DU145) irradiated with SBB

**Figure 10:**
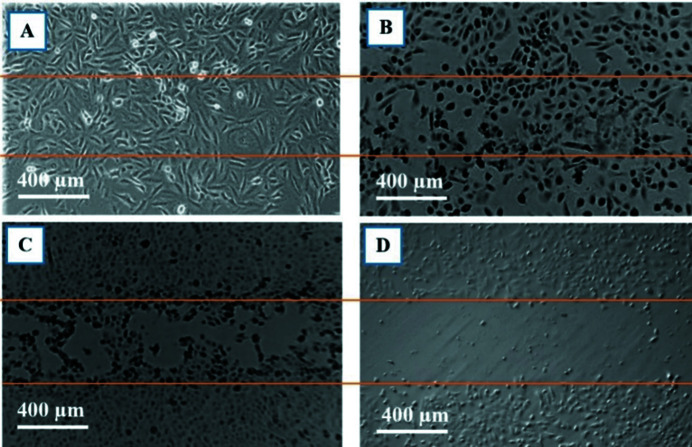
Human prostate cancer DU145 monolayer cells 24 h after being exposed to 500 µm SBB. The radiation fields are marked between orange lines. (A) 50 Gy, (B) 100 Gy, (C) 500 Gy and (D) 1000 Gy.

**Figure 11:**
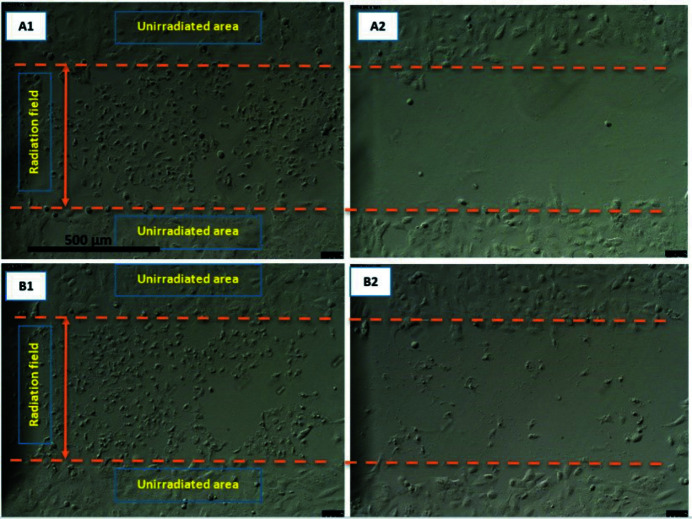
Human prostate DU145 cancer cells irradiated with 1000 Gy SBB. The radiation field is marked by orange dotted lines. (A1) Control (no AuNPs) 24 h, (A2) control (no AuNPs) 48 h, (B1) treated group (with 1 m*M* AuNPs) 24 h and (B2) treated group (with 1 m*M* AuNPs) 48 h post-irradiation with PBS wash.

A similar procedure was followed for human prostate (DU145) cancer cells. DU145 cells were irradiated with various doses, *i.e.* 50, 100, 500 and 1000 Gy. Based on the morphological changes of the cells after the exposure (Fig. 10 ▸), 1000 Gy was chosen to create a cell gap on the cells. As seen in Fig. 11 ▸, exposure of 1000 Gy SBB to DU145 in the control group (with no AuNPs) resulted in an almost clear cell gap 24 h post-irradiation. However, in the treated group (with 1 m*M* AuNPs) there are still some damaged cells attached to the flask surface.

### 3.3. Human epidermal melanocytes (HEM) and human primary melanoma (MM418-C1) irradiated with SBB

**Figure 12:**
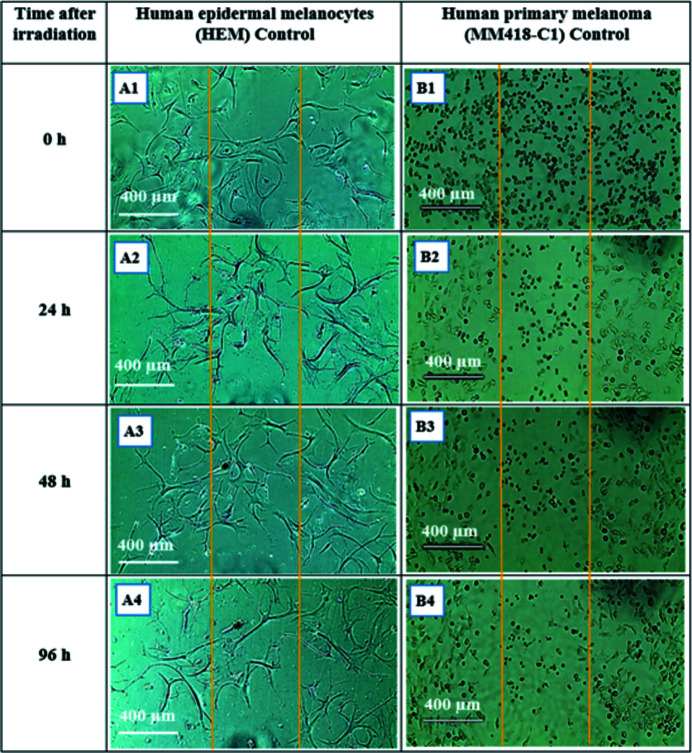
Human epidermal melanocytes HEM and human primary melanoma MM418-C1 monolayer 24 h after being exposed to 1000 Gy 90 kVp SBB (width 500 µm). Both cell types are control with no AuNPs treatment. The boundaries of the radiation fields are marked with orange lines. (A1) HEM immediately after exposure, (A2) 24 h, (A3) 48 h and (A4) 96 h after exposure. (B1) MM418-C1 immediately after exposure, (B1) 24 h, (B2) 48 h and (B4) 96 h after exposure.

A similar procedure as given in Section 3.1 was followed for human epidermal melanocyte (HEM) and its cancerous counterpart human primary melanoma (MM418). Both cell types were partitioned into the control (with no AuNPs) and those treated (with 1 m*M* AuNPs). Due to longer proliferation times for HEM, this observation was continued for 96 h post-irradiation. As seen in Figs. 12 ▸ and 13 ▸, MM418 cells located in radiation fields show visible morphological deformation in both control and treated groups such that the radiation field is clearly recognisable; its size is consistent with the actual radiation field which was 500 µm. Interestingly, HEM cells after exposure to 1000 Gy dose did not show any visible morphological damage in either the control (with no AuNPs) or those treated with 1 m*M* AuNPs groups.

### 3.4. Human primary colon epithelial (CCD841) and human colorectal adenocarcinoma (SW48) irradiated with SBB

**Figure 14:**
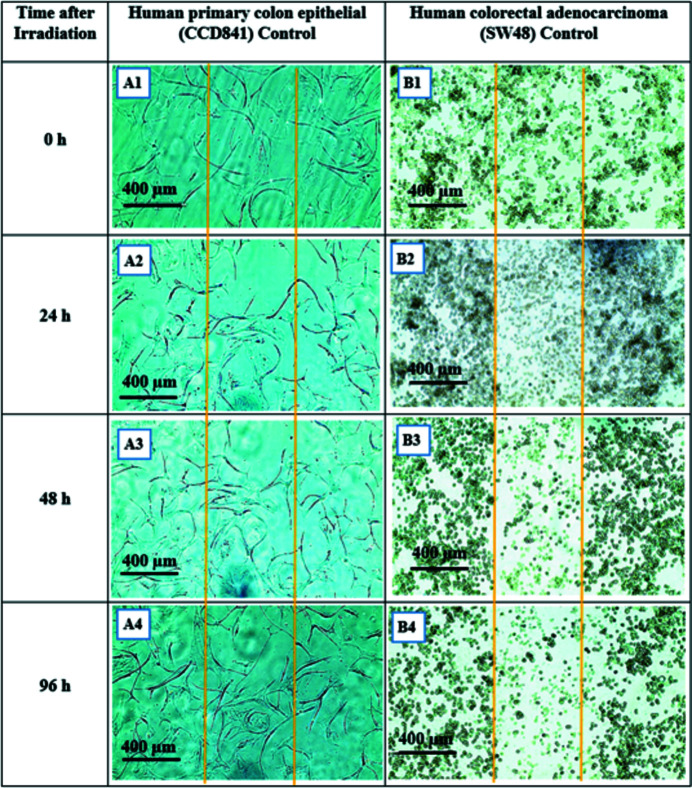
Human primary colon epithelial CCD841 and human colorectal adenocarcinoma SW48 monolayer 24 h after being exposed to 1000 Gy 90 kVp SBB (width 500 µm). Both cell types are control with no AuNPs treatment. The radiation fields boundaries are marked with orange lines. (A1) CCD841 immediately after exposure, (A2) 24 h, (A3) 48 h and (A4) 96 h after exposure. (B1) SW48 immediately after exposure, (B1) 24 h, (B2) 48 h and (B4) 96 h after exposure.

A similar procedure as given in Section 3.1 was followed for human primary colon epithelial (CCD841) and its cancerous counterpart, human colorectal adenocarcinoma (SW48). Both cell types were divided into the control with no AuNPs and those treated with 1 m*M* AuNPs. Due to longer proliferation times for CCD841 this observation was continued for 96 h post-irradiation. As seen in Figs. 14 ▸ and 15 ▸, SW48 cells located in radiation fields show visible morphological deformation in both the control and the treated groups such that the radiation field is clearly recognisable and its size is consistent with the actual radiation field which was 500 µm. Of interest was that CCD841 cells after exposure to 1000 Gy dose did not show any visible morphological damage in either the control (with no AuNPs) or those treated with 1 m*M* AuNPs groups.

A tabulated summary of the results for all experimental cell lines irradiated with SBB is given in Table 1 ▸.

### 3.5. Cell irradiation with SMB

A similar procedure to that described previously was used for irradiation with SMB (Section 3.1) to irradiate all six cell types, *i.e.* human prostate cancer (DU145), human lung cancer (A549), human primary melanoma (MM418), human colorectal adenocarcinoma (SW48), human epidermal melanocyte (HEM) and colon human primary colon epithelial (CCD8). The cells were irradiated with doses of 50, 500 and 1000 Gy (the maximum possible dose that can be delivered using the MRT collimator) with grid size of 175 µm (valley)/25 µm (peak). Follow-up microscopy observations up to 96 h post-irradiation did not show any visible deformation or morphology changes in any of the cell lines (either in the control or in the AuNPs treated groups).

## 4. Discussion

**Figure 16:**
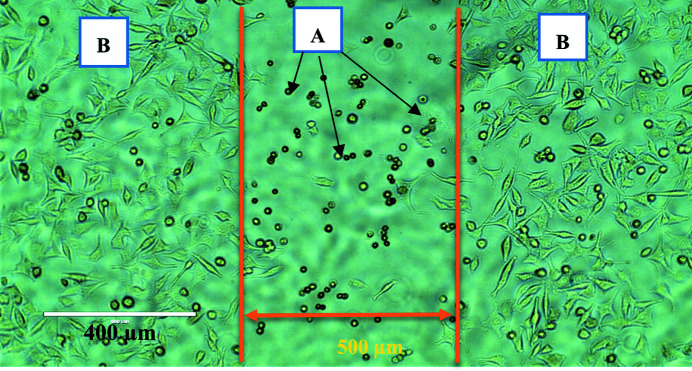
Morphological changes in MM418 cells 24 h after they were irradiated with 1000 Gy. The area enclosed between orange lines is exposed to 90 kVp SBB. (A) Apoptotic cells in irradiated area. (B) Viable cells in unirradiated area.

The main aim of this study was to investigate the effects of both SBB and SMB radiation on cancer and normal cell morphology and motility via *in vitro* based investigations. All cancer cell lines used in this study (DU145, A549, MM418 and SW48) showed a similar dose-dependent response to SBB. None of them showed any visible morphological deformation or changes after being exposed to up to 50 Gy doses. However, by increasing the dose to greater than 50 Gy, the cells located within the radiation field (500 µm) showed visible radiation-induced damage consistent with an apoptosis pattern, *i.e.* shrinking size and rounding shape with condensed cytoplasm (Larson & Banks, 2020 ▸). As seen in a phase contrast micrograph of MM418 (Fig. 16 ▸), after exposure to 1000 Gy dose, the cells within the radiation field 24 h post-exposure are demonstrating radiation-induced apoptosis.

Conversely, both normal cell lines used in our study, which were exposed to the same SSB and SMB dose range, demonstrate no visible radiation-induced morphological changes up to 1000 Gy. These cells were observed for longer than for the cancer cells (96 h) to ensure any late cell apoptosis was not missed. It should be noted here that doses in such very narrow fields are much less effective than in broad beams – for instance, 50 Gy of a broad beam is sufficient to kill almost all cancer cells.

Radiation-induced morphological changes in cancer and normal cells might be affected by their size compared with the radiation field size. It is known that cancer cells are normally presented in various sizes – they can be larger or smaller than normal cells (Eldridge, 2017 ▸).

**Figure 17:**
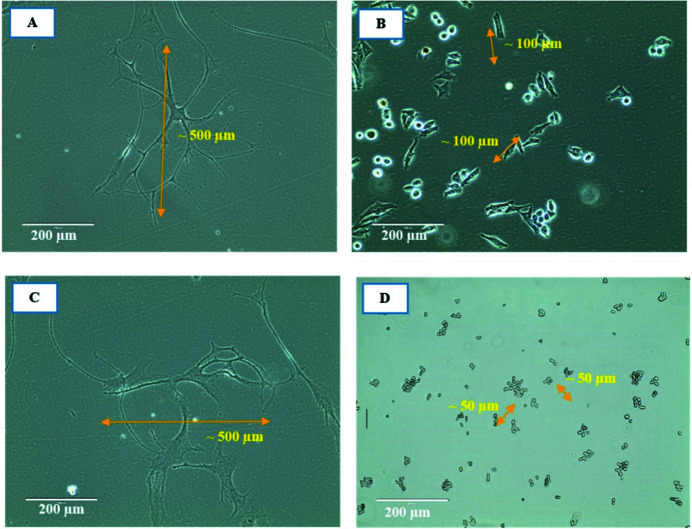
Phase-contrast 2D micrographs of HEM, MM418-C1, CCD841 and SW48. (A) HEM individual cell with average size/length of ∼500 µm, (B) MM418-C1 individual cells with average size/length of ∼100 µm, (C) CCD841 individual cell with average size/length of ∼500 µm and (D) SW48 individual cells with average size of ∼50 µm.

As seen in Fig. 17 ▸, there is a significant difference between cell sizes in normal cell lines, *i.e.* HEM and CCD841, and in the cancer cell lines, *i.e.* MM418 and SW48. Normal cell lines are about five times larger than their cancerous counterparts which may cause their different responses to the same radiation dose with the same radiation field size. The difference between the sizes of the cancer and normal cells and the radiation field size can be quantified by the following equation,

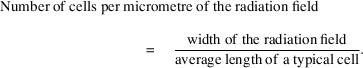

Therefore, the number of cells within a unit length of the radiation field can be estimated as follows. For HEM: (∼500)/500 ≃ 1; for MM418: (∼500)/100 ≃ 5; for CCD841: (∼500)/500 ≃ 1; for SW48: (∼500)/50 ≃ 10.

Based on the results, for normal cell lines (HEM and CCD841), on average, one cell can barely be covered or exposed in a 500 µm SBB radiation field and in the case of SMB this decreases to an even smaller fraction (∼10%) of a cell body. This is significantly smaller than the population of cancer cells that can be exposed in the same field area. Therefore, the hit probability in the case of the normal cell’s nucleus/DNA is lower due to the geometrical exposed cross section of the cells.

In addition, different responses to the radiation in the normal cells compared with the cancer cells can be attributed to the difference in cell-division cycle checkpoints amongst normal and cancer cells. The G1 phase is involved in cell growth and synthesis of the required proteins and S phase involved in DNA replication. A cell in the G2 phase enters further growth and then starts the M phase in which it undertakes cell division (Pawlik & Keyomarsi, 2004 ▸). A cell’s DNA is more radio-sensitive during the M (mitosis) phase and less at the end of the S and during the G2 phase. The normal cell lines (HEM and CCD841) that were used in this study exhibit longer doubling times (almost threefold longer) compared with their cancerous counterparts which means longer G1, S and G2 phases. Therefore, for these cells, there is a higher chance of being exposed during the most radio-resistant phases than for cancer cells.

This *in vitro* study showed that, under the same AuNPs treatment regimen and SBB radiation dose, lethal damage to cancer cells is significantly more pronounced compared with to normal cells, which highlights the promising role of synchrotron-based X-rays in future radiotherapy.

## Figures and Tables

**Figure 13:**
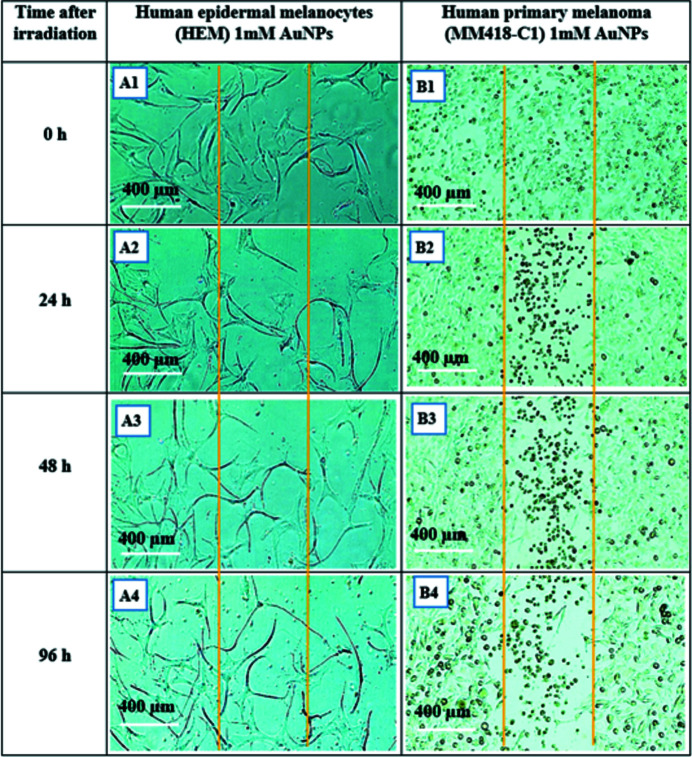
Human epidermal melanocytes HEM and human primary melanoma MM418-C1 monolayer 24 h after being exposed to 1000 Gy 90 kVp SBB (width 500 µm). Both cell types are treated with 1 m*M* AuNPs. The boundaries of the radiation fields are marked with orange lines. (A1) HEM immediately after exposure, (A2) 24 h, (A3) 48 h and (A4) 96 h after exposure. (B1) MM418-C1 immediately after exposure, (B1) 24 h, (B2) 48 h and (B4) 96 h after exposure.

**Figure 15:**
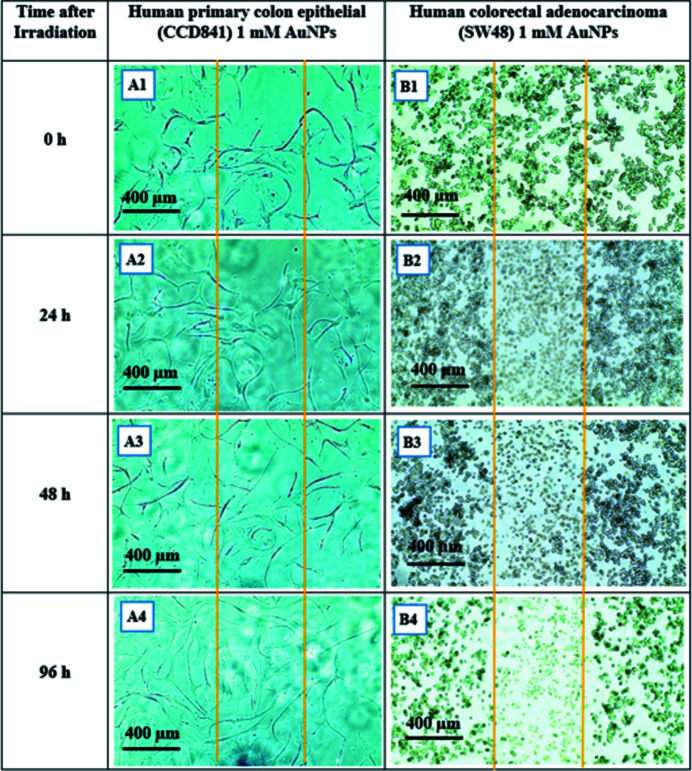
Human primary colon epithelial CCD841 and human colorectal adenocarcinoma SW48 monolayer 24 h after being exposed to 1000 Gy 90 kVp SBB (width 500 µm). Both cell types are treated with 1 m*M* AuNPs. The radiation fields boundaries are marked with orange lines. (A1) CCD841 immediately after exposure, (A2) 24 h, (A3) 48 h and (A4) 96 h after exposure. (B1) SW48 immediately after exposure, (B1) 24 h, (B2) 48 h and (B4) 96 h after exposure.

**Table 1:** A summary of the results of microscopic observation 24 h post-irradiation of DU145, A549, HEM, MM418, CCD841 and SW48 in control and treated groups irradiated with various doses of SBB

Cell type	SBB dose (Gy)	Experimental groups	Morphological changes within the exposed area	Cell gap created
DU145 (prostate cancer)	50	Control	No	No
		1 m*M* AuNPs	No	No
	100	Control	∼10%	No
		1 m*M* AuNPs	∼10%	No
	500	Control	∼50%	No
		1 m*M* AuNPs	∼50%	No
	1000	Control	Almost all cells	Yes
		1 m*M* AuNPs	Almost all cells	Yes
A549 (lung cancer)	50	Control	No	No
		1 m*M* AuNPs	No	No
	100	Control	∼10%	No
		1 m*M* AuNPs	∼10%	No
	500	Control	∼50%	No
		1 m*M* AuNPs	∼50%	No
	1000	Control	Almost all cells	No
		1 m*M* AuNPs	Almost all cells	No
EM (skin normal)	50	Control	No	No
		1 m*M* AuNPs	No	No
	100	Control	No	No
		1 m*M* AuNPs	No	No
	500	Control	No	No
		1 m*M* AuNPs	No	No
	1000	Control	No	No
		1 m*M* AuNPs	No	No
MM418 (skin cancer)	50	Control	No	No
		1 m*M* AuNPs	No	No
	100	Control	∼10%	No
		1 m*M* AuNPs	∼10%	No
	500	Control	∼50%	No
		1 m*M* AuNPs	∼50%	No
	1000	Control	Almost all cells	No
		1 m*M* AuNPs	Almost all cells	No
CCD841 (colon normal)	50	Control	No	No
		1 m*M* AuNPs	No	No
	100	Control	No	No
		1 m*M* AuNPs	No	No
	500	Control	No	No
		1 m*M* AuNPs	No	No
	1000	Control	No	No
		1 m*M* AuNPs	No	No
SW48 (colon cancer)	50	Control	No	No
		1 m*M* AuNPs	No	No
	100	Control	∼10%	No
		1 m*M* AuNPs	∼10%	No
	500	Control	∼50%	No
		1 m*M* AuNPs	∼50%	No
	1000	Control	Almost all cells	No
		1 m*M* AuNPs	Almost all cells	No
